# Different endocytotic uptake mechanisms for nanoparticles in epithelial cells and macrophages

**DOI:** 10.3762/bjnano.5.174

**Published:** 2014-09-24

**Authors:** Dagmar A Kuhn, Dimitri Vanhecke, Benjamin Michen, Fabian Blank, Peter Gehr, Alke Petri-Fink, Barbara Rothen-Rutishauser

**Affiliations:** 1 Adolphe Merkle Institute, University of Fribourg, Chemin des Verdiers 4, 1700 Fribourg, Switzerland, Phone +41 26 300 95 02; 2 Respiratory Medicine, University Hospital of Bern, Murtenstrasse 50, 3008 Bern, Switzerland; 3 Institute of Anatomy, University of Bern, Baltzerstrasse 2, 3000 Bern 9, Switzerland

**Keywords:** cell lines, endocytosis, inhibition, nanoparticles, uptake proteins

## Abstract

Precise knowledge regarding cellular uptake of nanoparticles is of great importance for future biomedical applications. Four different endocytotic uptake mechanisms, that is, phagocytosis, macropinocytosis, clathrin- and caveolin-mediated endocytosis, were investigated using a mouse macrophage (J774A.1) and a human alveolar epithelial type II cell line (A549). In order to deduce the involved pathway in nanoparticle uptake, selected inhibitors specific for one of the endocytotic pathways were optimized regarding concentration and incubation time in combination with fluorescently tagged marker proteins. Qualitative immunolocalization showed that J774A.1 cells highly expressed the lipid raft-related protein flotillin-1 and clathrin heavy chain, however, no caveolin-1. A549 cells expressed clathrin heavy chain and caveolin-1, but no flotillin-1 uptake-related proteins. Our data revealed an impeded uptake of 40 nm polystyrene nanoparticles by J774A.1 macrophages when actin polymerization and clathrin-coated pit formation was blocked. From this result, it is suggested that macropinocytosis and phagocytosis, as well as clathrin-mediated endocytosis, play a crucial role. The uptake of 40 nm nanoparticles in alveolar epithelial A549 cells was inhibited after depletion of cholesterol in the plasma membrane (preventing caveolin-mediated endocytosis) and inhibition of clathrin-coated vesicles (preventing clathrin-mediated endocytosis). Our data showed that a combination of several distinguishable endocytotic uptake mechanisms are involved in the uptake of 40 nm polystyrene nanoparticles in both the macrophage and epithelial cell line.

## Introduction

In recent years, the use of engineered nanoparticles (NPs) (defined as <100 nm in three dimensions according to ISO TS 27687:2008) has witnessed a strong rise in biomedical and pharmaceutical applications, specifically for targeted drug delivery [1–6], biosensing [7] and bio-medical imaging [8]. In order to develop optimal NPs for biomedical use, much attention is given to the understanding of the basic mechanism of NP interactions with cellular systems at the single cellular level [9–11]. It has already been shown that different NP properties, such as size, shape, material and surface coating, as well as the cell type, age, interaction with other cells and the cellular environment, influence NP uptake and the cellular behavior as well as the down-stream response of the cells [11–16].

The term endocytosis describes two different cellular uptake mechanisms: pinocytosis, which involves the uptake of fluids and molecules within small vesicles and phagocytosis, which is responsible for engulfing large particles (e.g., microorganisms and cell debris). Pinocytosis covers macropinocytosis, clathrin-mediated endocytosis, caveolin-mediated endocytosis and clathrin- and caveolin-independent endocytosis [8,17–20]. However, not all cell types are equipped with the required machinery to perform the entire spectrum of endocytotic pathways. Therefore, these pathways are specific to types of cells and subsequently determine the trafficking and intracellular fate of particles [21]. Red blood cells are a common example, as they do not have any phagocytic receptors on their surface and no actin–myosin system, therefore they serve as a model for non-phagocytic cells to study how NPs penetrate through cell membranes [22].

Phagocytosis and macropinocytosis are both dependent on actin [15,23]. Phagocytosis is carried out by professional phagocytes (i.e., monocytes/macrophages, neutrophils and dendritic cells), which in turn form intracellular phagosomes. Macromolecule and particle uptake is triggered via the interaction of the responsible receptors on the cell surface and the ligands. Macropinocytosis, which is also actin-driven, forms protrusions at the outer cell membrane which then again fuse with the cell membrane by taking up larger fragments or debris [14].

Clathrin-mediated endocytosis is very well studied and is, like most pinocytotic pathways, a form of receptor-mediated endocytosis. This abundant pathway is essential for the uptake of many molecules such as low-density lipoprotein and transferrin [24–25]. When clathrin-mediated endocytosis is initiated, the so-called “coated pits” come into play consisting of transmembrane receptors and cytosolic proteins, such as clathrin and the AP2 adaptor complex [20].

On the other hand, caveolin-mediated endocytosis is responsible for the homeostasis of cholesterol [20]. The static structures of caveolae form flask-shaped invaginations in the cell membrane. Many cell types such as the capillary endothelium, type I epithelial cells, muscle cells as well as fibroblasts, exhibit caveolin-mediated endocytosis, which occurs at the site of the lipid rafts [20,26]. These rafts are plasma membrane regions (subdomains), which consist of glycosphingolipids and high amounts of cholesterol [27]. The protein which gives shape and structure in caveolin-mediated endocytosis is caveolin-1, a dimeric protein which binds cholesterol onto the cellular surface for uptake and intracellular trafficking (lipid homeostasis) [28]. Also located at the site of lipid rafts is flotillin-1, an integral membrane protein which forms a hetero-oligomer with flotillin-2 [29]. In addition to the aforementioned uptake mechanisms, clathrin- and caveolin-independent endocytosis as well as passive diffusion of NPs across the cell plasma membrane are also addressed [17,30].

To elaborate on the most important cellular endocytotic uptake mechanism of NPs, specific pharmacological substances which inhibit specific pathways can be used [31]. It is important to highlight that the use of inhibitors must be optimized for each cell and NP type, since an inhibitor might show a high specificity in one experiment but cause side effects in another [32]. The use of positive controls to show that an inhibitor only affects one endocytotic pathway without interfering with other uptake mechanism(s) is mandatory [33]. There are many different inhibitors described, and we will focus only on the most commonly used drugs to study NP uptake.

Cytochalasin D can depolymerize actin filaments [34–35] and can therefore be used to study actin-dependent uptake mechanisms, that is, phagocytosis and macropinocytosis. Larger particles, such as polystyrene particles of 1 µm in diameter, can be used to run the experiment under controlled conditions.

Chlorpromazine hydrochloride which inhibits clathrin-mediated endocytosis, induces a loss of clathrin and adaptor protein complex 2 from the surface of the cell [31,36]. It is thus classified as an inhibitor for clathrin-mediated endocytosis [37–38]. Monodansylcadaverine (MDC), a competitive inhibitor, blocks the enzyme transglutaminase 2, which is necessary for receptor crosslinking in the region of clathrin-coated pits [31,39–40]. Furthermore, chlorpromazine and MDC are specific in inhibiting the uptake of the serum protein transferrin [41]. Consequently, fluorescently labelled transferrin can be used to investigate clathrin-mediated endocytosis [32,41–42].

Caveolae and lipid raft internalizations are known to be inhibited by nystatin, filipin and methyl-β-cyclodextrin (mβcd) through depletion of the cholesterol from the cell membrane by forming inclusion complexes with cholesterol [31,43]. It was also shown that mβcd inhibits clathrin-mediated endocytosis, since clathrin uptake requires cholesterol as well [11,44]. All of these mentioned inhibitors form aggregates which accumulate cholesterol and separate it from the membrane structures. Finally, the cholera toxin subunit b (ctx-b) [45] has been shown to enter the cells by caveolin-mediated endocytosis, therefore this protein can be used to control the inhibitors in any experimental setting.

To show that the endocytotic uptake route of choice is dependent upon the particle size, we deployed two fluorescently labelled polystyrene particles of significantly different sizes, that is, 40 nm and 1 µm in diameter. These particles were chosen as they are easy to detect by fluorescence methods and available in different sizes [46]. Moreover, they have a narrow size distribution and are considered to be suitable for biomedical applications [47–48] since they are considered non-toxic at applied physiological concentrations [49]. Finally, two of the most relevant cell types in regard to uptake and interaction of (nano) particles at any barrier system (i.e., macrophages and epithelial) [50–51] were included to demonstrate that not only the applied particle dimensions and uptake pathways are determinant, but the actual cell types as well.

## Results

### Particle characterization

**Figure 1:**
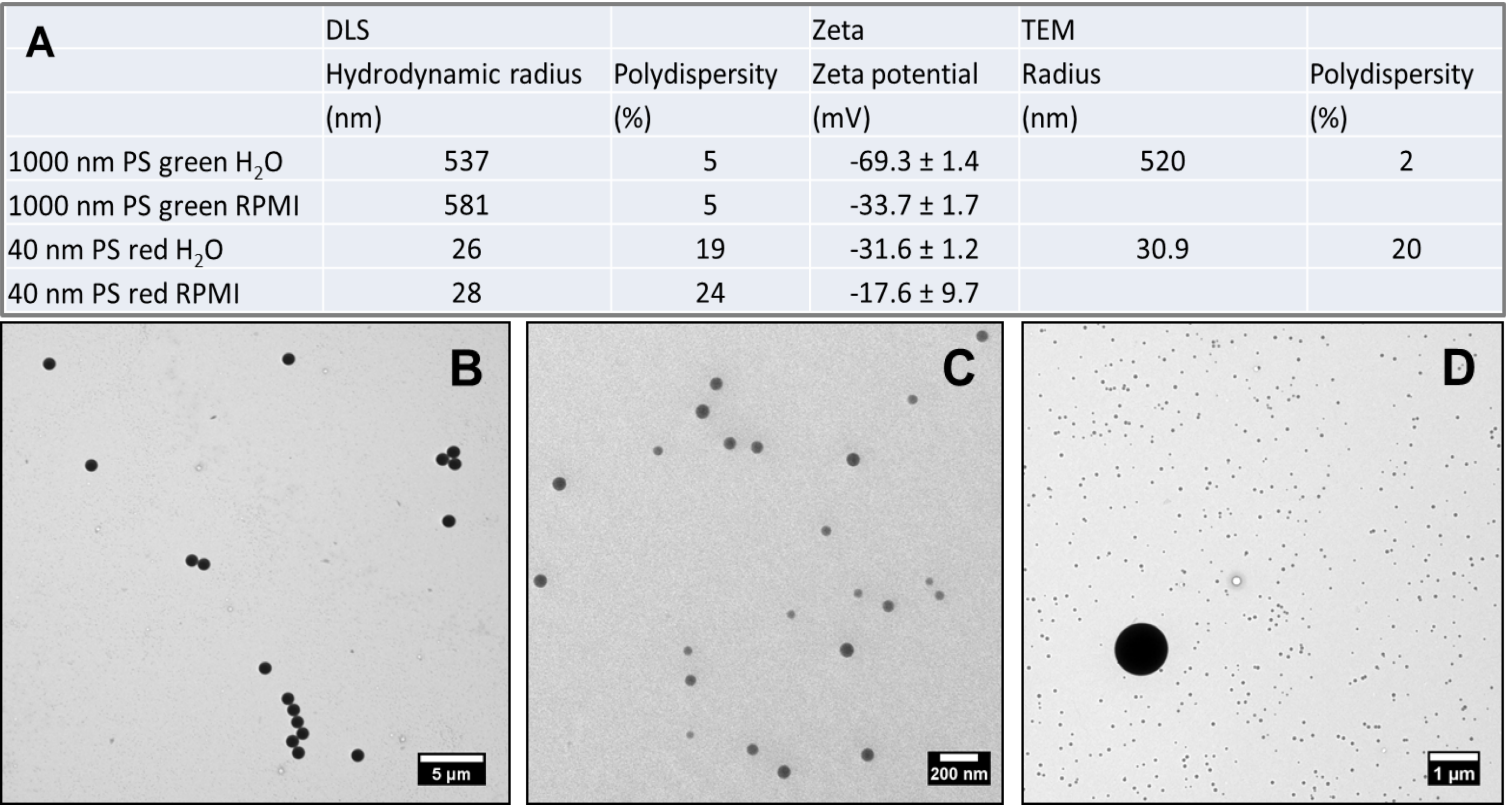
Characterization of polystyrene particles. (A) Characteristics of the particles as measured by dynamic light scattering, zeta potential and transmission electron microscopy in water and unsupplemented cell culture medium. Transmission electron microscopy images of (B) 1 µm particles, (C) NPs, and (D) a mixture of 1 µm particles and NPs.

In the first step, both particles were thoroughly investigated prior to cellular experiments. The particle size measured with dynamic light scattering (DLS) revealed an average hydrodynamic radius of approximately 581 nm for the microparticles and 28 nm for the nanoparticles. Transmission electron microscopy (TEM) revealed a core radius of 520 nm for the microparticles and 30.9 nm for the NPs. The latter, however, exhibited a larger polydispersity (Figure 1). Both methods confirm the experimentally obtained values. Measurements were carried out in water and unsupplemented RPMI medium and showed that all analyzed particles remained stable and monodisperse in biological medium. The zeta potential indicated a negative charge for both particle types which was slightly reduced, but still negative, when the particles were suspended in unsupplemented RPMI medium (Figure 1).

### Expression of endocytotic uptake proteins in both cell types

**Figure 2:**
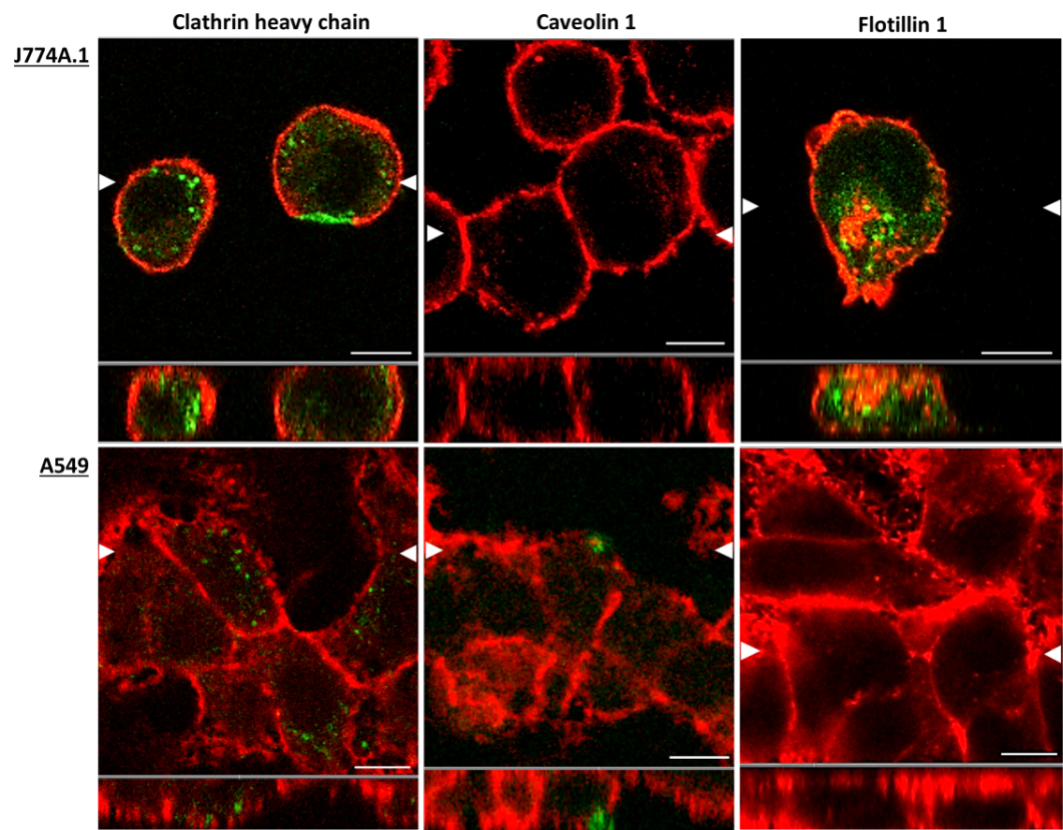
LSM images demonstrate the presence of the different endocytotic uptake proteins within J774A.1 macrophages and A549 epithelial cells. The LSM images show the presence of endocytotic proteins clathrin heavy chain, caveolin-1 and flotillin-1 in J774A.1 cells and A549 cells. The white arrows in the upper panel of each image (XY orthogonal plane) represent the position of the XZ slice shown in the lower picture. The apical side of the cells corresponds to the bottom line of the images. Endocytotic uptake proteins are shown in green and the actin cytoskeleton in red. Scale bar: 8 µm.

In order to define particle uptake routes, it is crucial in the first step to determine the presence of the endocytotic proteins which are involved in endocytosis in both cell types (Figure 2). To achieve this, laser scanning microscopy (LSM) was applied as the primary tool for this investigations. Flotillin-1 and clathrin heavy chain could be visualized in J774A.1 cells, but caveolin-1 was not detected. Clathrin heavy chain was detected within the cells, both at the cell membrane and in the cytosol. Flotillin-1 was, however, only observed in the cytosol. In A549 cells, clathrin heavy chain and caveolin-1 were located in the cells both at the cell membrane as well as in the cytoplasm, albeit to a lower extent than in the macrophages. Flotillin-1 could not be detected in A549 cells.

### Endocytotic inhibitors

**Figure 3:**
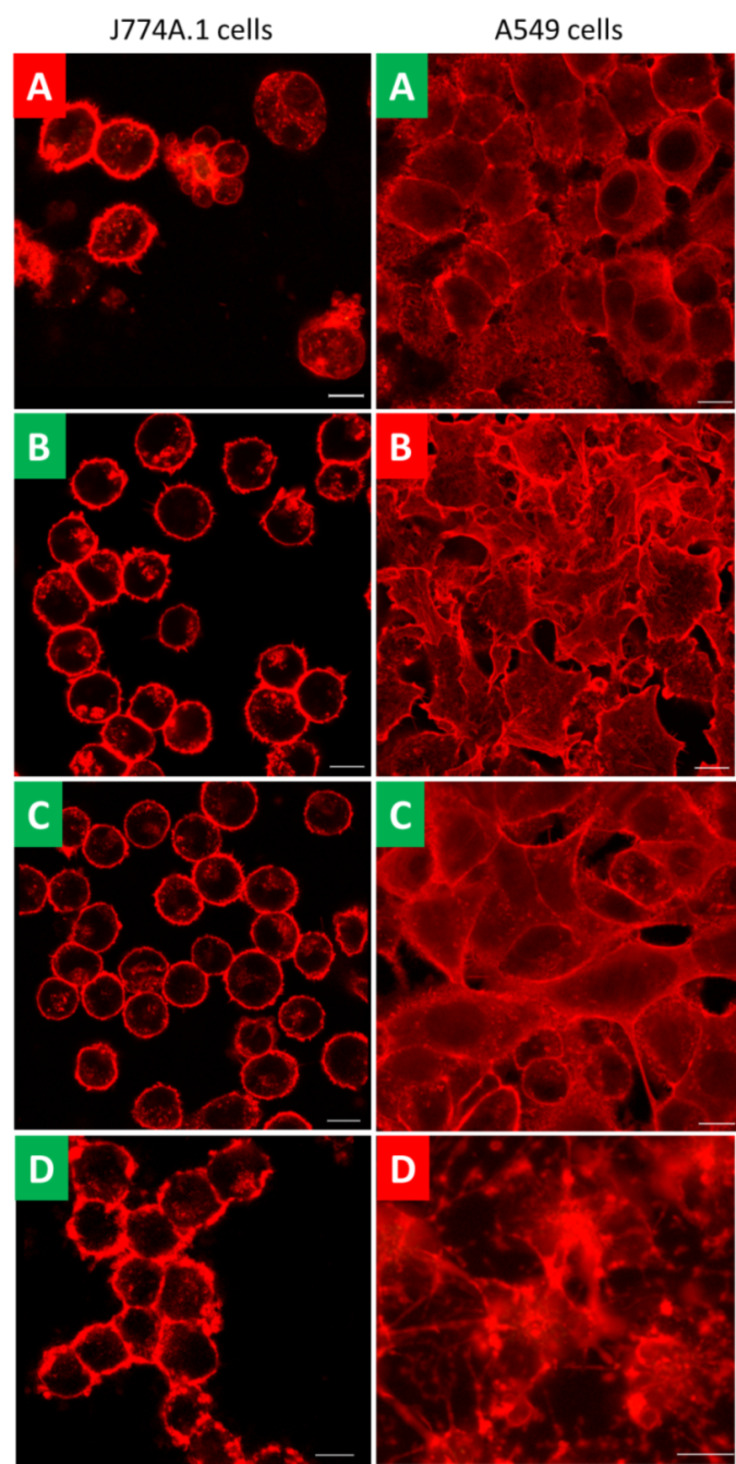
Investigation of cell morphology after inhibitor treatment. Healthy cells (green inset) retained their cellular structure after inhibitor treatment. Impaired cells (red inset) showed membrane damage, loss of integrity and loss of viability. (A) chlorpromazine, (B) monodansylcadaverine, (C) mβcd, and (D) cytochalasin D. Scale bar: 10 µm.

With the knowledge of the presence of specific uptake proteins, it is now feasible to elaborate on the different uptake routes in each cell type by applying different chemical inhibitors. Inhibitors of clathrin- and caveolin-mediated endocytosis or macropinocytosis and phagocytosis were tested for their optimal concentration, exposure time and cell impairment in both cell types (Table 1 and Figure 3). The cell morphology was assessed by LSM (Figure 3) and the cytotoxicity by lactate dehydrogenase (LDH) assay (see Figure S1, Supporting Information File 1). Trypan blue staining marked the integrity of the cell membrane for cells which were impaired by the inhibitor (red insets, Figure 3) and revealed the following percentage of dead cells for J774A.1 cells (*n* = 3): negative control 20% (SD ± 9.5%), triton 100% (SD ± 0%), chlorpromazine 46.1% (SD ± 2.7%). For A549 cells, the following percentages of dead cells were revealed: negative control 0% (SD ± 0%), triton 100% (SD ± 0%), MDC 78.3% (SD ± 15.5%), cytochalasin D 10.3% (SD ± 9.6%). Fluorescently labelled transferrin was used together with specific inhibitors as a control to investigate clathrin-mediated endocytosis and fluorescent ctx-b with inhibitors as a control to analyze caveolin-mediated endocytosis. Polystyrene particles of 1 µm diameter were used to demonstrate the inhibition of phagocytosis. The uptake of fluorescently labelled transferrin was blocked by applying 250 µM MDC in J774A.1 cells, whereas MDC could not inhibit clathrin-mediated endocytosis in A549 cells. In addition, treatment of A549 cells with MDC resulted in cell impairment as shown by LSM (Figure 3). Chlorpromazine did not inhibit transferrin uptake and also severely impaired the morphology of the J774A.1 cells. However, 100 μM chlorpromazine inhibited the clathrin-mediated endocytosis of A549 cells. Mβcd could neither inhibit transferrin (clathrin-mediated) nor ctx-b (caveolin-mediated) uptake by J774A.1 macrophages. On the contrary, 10 mM mβcd inhibited clathrin- as well as caveolin-mediated endocytosis as shown by the lack of intracellular fluorescently labelled transferrin and ctx-b in A549 cells. The actin polymerization inhibitor cytochalasin D prevented particle uptake (*d* = 1 µm) by J774A.1 cells at a concentration of 4 µM. Uptake of transferrin by J774A.1 cells could not be prevented by cytochalasin D (data not shown). In A549 cells, cytochalasin D impaired the cell morphology at all tested concentrations from 5 µM to 10 µM. A lower concentration of 3 µM did not inhibit particle uptake (data not shown). It is important to mention that inhibition of cells with cytochalasin D cannot distinguish between phagocytosis and macropinocytosis.

**Table 1:** Analysis of the different endocytotic inhibitors regarding specificity and efficiency.

***J774A.1* cells**						
Inhibitors	*c* (µM)	Exposure (*t*)	Transferrin	Ctx-b	1 µm PS	Cell morph.^a^

Chlorpromazine (clathrin)	100	30 min	No inhibition	No inhibition	No inhibition	–
MDC (clathrin)	250	1 h 30 min	**Inhibition**	No inhibition	No inhibition	+
MβCD (clathrin, caveolin)	10·10^3^	30 min	No inhibition	No inhibition	No inhibition	+
CytoD (phag., macrop)	4	1 h 30 min	No inhibition	No inhibition	**Inhibition**	+

***A549* cells**						
Inhibitors	*c* (µM)	Exposure (*t*)	Transferrin	Ctx-b	1 µm PS	Cell morph.^a^

Chlorpromazine (clathrin)	100	30 min	**Inhibition**	No inhibition	No inhibition	+
MDC (clathrin)	250	1 h 30 min	No inhibition	No inhibition	No inhibition	–
MβCD (clathrin, caveolin)	10·10^3^	30 min	**Inhibition**	**Inhibition**	No inhibition	+
CytoD (phag., macrop.)	4	1 h 30 min	No inhibition	No inhibition	No inhibition	–

^a^Cell morphology: no cellular impairment (+) and cellular damage (-).

### Fluorescence intensity profiles

**Figure 4:**
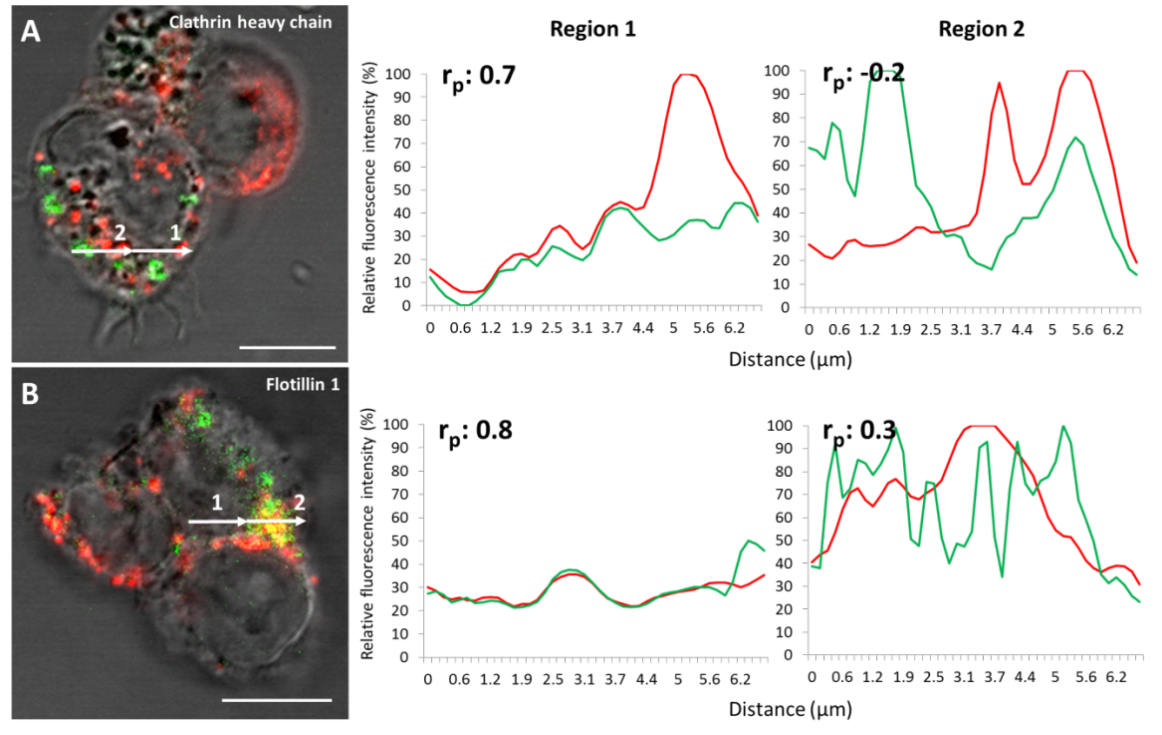
Fluorescence intensity profiles of J774A.1 cells, 40 nm PS NPs with clathrin heavy chain or flotillin-1. (A) and (B) are confocal images of J774A.1 cells treated with 40 nm PS NPs (red) and primary antibodies against uptake proteins (green), respectively. The corresponding relative fluorescence profiles of the overlapping signals are shown in the diagrams to the right (regions 1 and 2). Cells are shown in the transmission light channel. The Pearson coefficient (r_p_) was calculated for each of the regions. (A) 40 nm PS NPs and clathrin heavy chain, (B) 40 nm PS NPs and flotillin-1. The white arrows are 6.8 µm in length. Scale bar = 10 µm.

To identify in which protein uptake compartments the NPs (*d* = 40 nm) are present, it is possible to analyze their regions of fluorescence overlap. Several intensity profiles reveal a distinct overlap (Figure 4A) of clathrin heavy chain and a signal from 40 nm NPs in J774A.1 cells (Figure 4A, region 1). However, cases of dissimilarities between the two fluorescence signals were also recorded (Figure 4A, region 2). Analogous observations (both in agreement and disagreement) of the flotillin-1 fluorescence signal and 40 nm PS NPs were made (Figure 4B, regions 1 and 2). These findings were supported by the resulting Pearson coefficient value found for each region analyzed. Intensity profile plots of subcellular events in A549 cells were not performed due to a lower expression of the uptake proteins in these cells compared to the J774A.1 cells.

### Particle uptake by the two cell types in the presence of endocytotic uptake inhibitors

**Figure 5:**
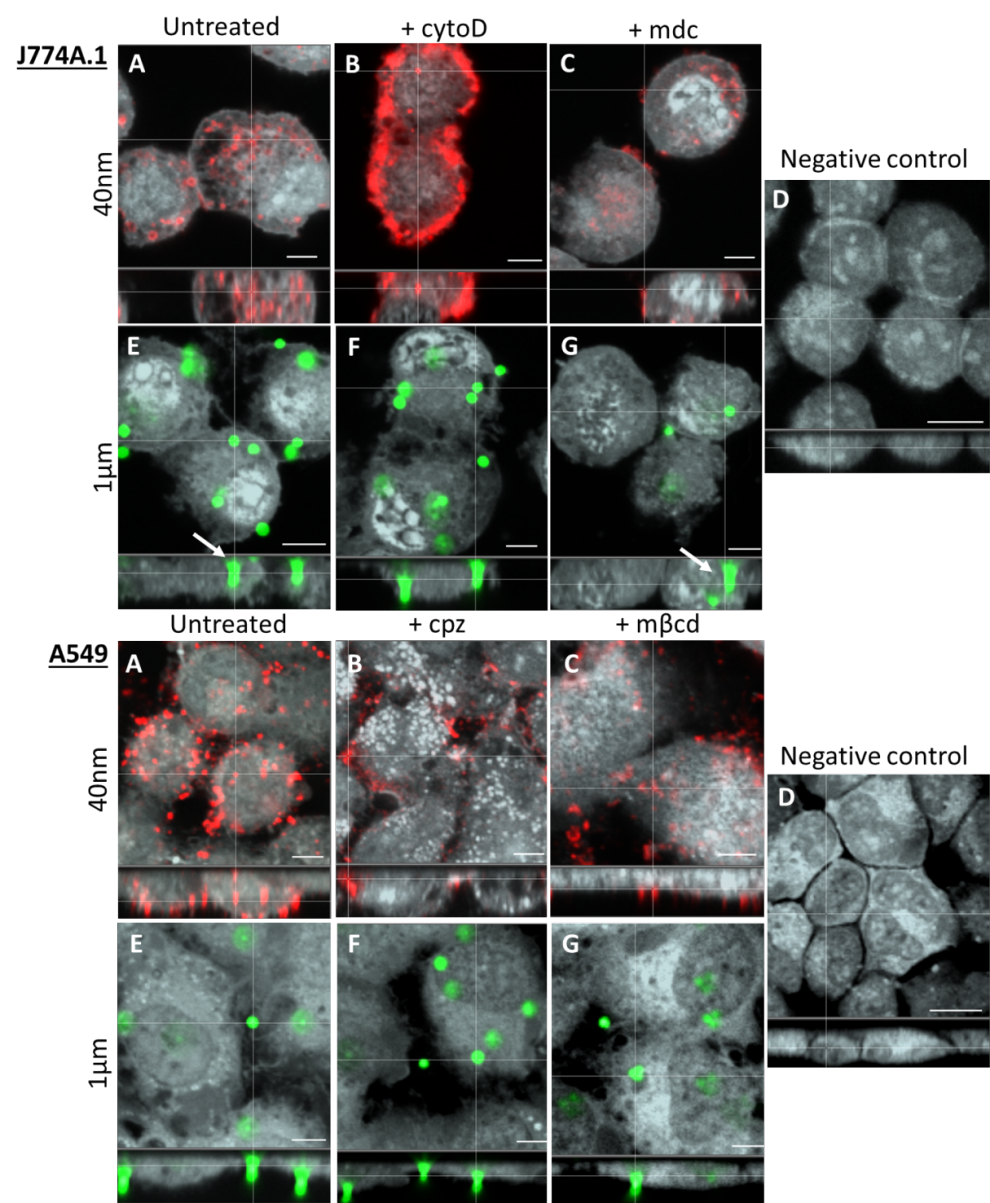
Laser scanning microscopy imaging revealed particle uptake in J774A.1 and A549 cells. (A–C) Uptake of 40 nm PS NPs (NP: red, cytosol: grey). (A) Untreated cells with 40 nm NPs. (B) 40 nm NPs and cytochalasin D (cytoD) in J774A.1 and chlorpromazine (cpz) in A549 cells. (C) 40 nm NPs and monodansylcadaverine (mdc) in J774A.1 cells and methyl-β-cyclodextrin (mβcd) in A549 cells. (D) Negative control. (E–G) Uptake of 1 µm PS particles (particles: green, cytosol: grey). (E) Untreated cells with 1 µm particles. (F–G) 1 µm particles with the same adequate inhibitors. Scale bar: 5 µm. White arrows represent intracellular events of 1 µm particles in J774A.1 cells. The negative controls have a scale bar of 10 µm.

In order to resolve the uptake routes for the given particle size, both particles were tested together with the optimal inhibitor concentration for both cell types. Conditions were chosen such that the uptake of the relevant control substance was completely inhibited and no impaired cell morphology was observed. Both cell lines were exposed to either 1 µm PS particles or 40 nm PS NPs at a concentration of 20 µg/mL for 1 hour either after preincubation with endocytotic inhibitors (preinhibition experiment) or in coexposure (continuous experiment) with endocytotic inhibitors (chlorpromazine, MDC, mβcd, cytochalasin D or none). Intracellular particles were visualized by LSM (Figure 5).

### Particle uptake evaluation

After 1 hour of incubation, intracellular particles and NP events (either agglomerates or single NPs, which cannot be distinguished by LSM) were visualized in both J774A.1 and A549 cells (Figure 5 and Figures S2 and S3, Supporting Information File 1). In control cells that were not treated with any inhibitors, uptake of NPs was observed within only a few minutes in both cell types (Figure 5). These observations are supported by live cell imaging, which revealed that NP uptake is a very fast process, starting 5 to 10 minutes after exposure to the cells (Figures S2 and S3, Supporting Information File 1). Uptake of 1 µm particles was only observed in J774A.1 macrophages but not in A549 epithelial cells for the given exposure time of 1 hour. A549 cells required a much longer time of 1 hour to internalize 1 µm particles (data not shown). However, since the inhibitors began to induce cell damage after 1 to 1.5 hours, observation time could not be extended. In J774A.1 cells, MDC partially inhibited the uptake of 40 nm NPs (Figure 5). A significant fraction of the 40 nm particles was observed outside at the cell membrane (Figure S2, Supporting Information File 1). However, NPs were also detected within the cells (Figure 5). Cytochalasin D also partially blocked the uptake of NPs. The uptake of 1 µm particles by J774A.1 was completely blocked by cytochalasin D, whereas MDC had no effect.

Although most 40 nm NP events were located outside of the cells, intracellular particle events could still be detected in both chlorpromazine-treated and mβcd-treated A549 cells. The 1 µm particles were not observed inside the epithelial cells under any condition (Figure 5).

## Discussion

For any future biomedical application of engineered NPs, it is mandatory to fundamentally understand their interaction with living systems. The cellular uptake pathway of a NP will have direct consequences on its intracellular localization; hence, understanding the overall NP distribution in a specific compartment, such as endosomes, lysosomes or others, might provide some interesting suggestions for developing a future drug delivery system. To gain more insight into the uptake mechanism(s) of NPs in comparison to larger (i.e., micron-size) particles of the same material, a broad array of chemical inhibitors was used that were shown to inhibit certain endocytotic mechanisms [52–54]. All particle exposure experiments were conducted in serum-free medium in order to avoid binding of serum proteins to the particle surfaces which might induce agglomeration, however, the binding of small molecules or salts in the cell culture medium cannot be excluded.

The focus of the present study was on two cell types that have important clearing and barrier functions, namely, macrophages (represented by the mouse J774A.1 macrophage cell line) and epithelial cells (represented by the A549 human alveolar epithelium cell line). First, the specificity of the different inhibitors was assessed for both cell types. As a second step, the optimized inhibitors were used to study the uptake of the two different particle types. Visualization of the fluorescently tagged particles was done by LSM. LDH measurements revealed no cytotoxicity for the combined inhibitors and endocytotic protein markers for both cell types being analyzed. Additionally, inhibitors which negatively affected the cells are summarized in Table 1 and the images in Figure 3 are presented with either red (impairment by inhibitor) or green (no effect by inhibitor) letter insets. Trypan blue staining demonstrated the same outcome. The percentage of dead cells which were treated with cytochalasin D was not as high as expected for A549 cells. This could be due to cytochalasin D blocking actin polymerization and hence preventing Trypan blue from entering the cells, although the cells are severely affected by this inhibitor.

It was shown that the optimal and desired function of the endocytotic inhibitors was occasionally different in the two cell types, namely, the A549 epithelial cells and J774A.1 macrophages. The inhibition studies were carried out within 1 hour, since endocytotic processes were very fast [20,55–56] and the inhibitors also began to impair the cells in their morphology after a longer incubation time. For each of the two cell types, the exposure time and concentration to achieve optimal inhibition had to be defined. This careful optimization for each inhibitor and each cell type is very often missing in many published studies. This balancing act between the desired functional inhibition of a specific pathway and the possible adverse effects on the cells did not always provide an applicable result. For instance, for some of the inhibitors the concentrations had to be very high for an efficient inhibition but then in this case they strongly impaired the cells. mβcd also inhibited the uptake of transferrin (clathrin-mediated endocytosis) in A549 cells [57], however, mβcd had detrimental effects on the J774A.1 cell morphology and could only be exposed for a limited period of 30 minutes preincubation [32].

In J774A.1 macrophages, macropinocytosis and phagocytosis could be optimally inhibited by cytochalasin D and MDC which could efficiently block clathrin-mediated endocytosis. However, optimal conditions for the inhibition of the lipid raft-mediated pathway in this cell type could not be established. mβcd has been described to be an optimal inhibitor of lipid raft-mediated endocytosis. Therefore it was rather surprising that it did not work in the J774A.1 cells, also because flotillin-1 was highly expressed in all cells as shown by LSM. However, the localization of the protein was mainly intracellular and caveolin-1 was not present at all. This finding should be evaluated more thoroughly. In addition, other inhibitors such as statins, filipin or nystatin could be used, however, severe cell damage for those three inhibitors has been observed in earlier studies [32].

In A549 epithelial cells, the macropinocytosis and phagocytosis pathways could not be explored, as the uptake of 1 µm particles by these cells was never observed, even under controlled conditions. Surprisingly, these findings are in contradiction to other studies that have shown uptake of such particles [30,58]. However, these A549 cells were used in a triple cell coculture system combined with macrophages and dendritic cells. Therefore, the results are not directly comparable. In addition, the uptake was evaluated only within 1 hour, and phagocytosis/macropinocytosis might be slower in epithelial cells than in macrophages. Therefore, longer incubation times should be applied in future studies, but also at optimal conditions under which the inhibitors do not induce adverse cell effects. The clathrin-mediated endocytosis was impeded by 100 μM chlorpromazine. Also, 10 mM mβcd was shown to block caveolin-mediated endocytosis with the restriction of a maximum of 30 minutes exposure in the inhibitor. Consequently, this cell line seems to be capable of ingesting particles by clathrin- as well as caveolin-mediated endocytosis. This is underlined by the presence of clathrin heavy chain and caveolin-1 in the cytoplasm and at the cell membrane. Interestingly, these cells do not show flotillin-1 compared to the J774A.1 cells. Nonetheless, the role of this protein on NP uptake will need to be further evaluated in the future.

It is obvious from these results that the inhibitors have different effects depending on the cell type. The reasons for this observation can be many: the genotypic and phenotypic differences between the cell types as well as species differences (i.e., the macrophages are a mouse cell line, while the epithelial cells are human) play a role. Hence, a preliminary study to optimize the use of inhibitors with each cell type should be a prerequisite for every future investigation involving endocytotic pathway inhibitors. To summarize, one can say that each cell type reacts differently to the applied inhibitors and possess different uptake routes.

Regarding the (nano) particle uptake study, cytochalasin D completely blocked the uptake of 1 µm particles via phagocytosis and macropinocytosis in J774A.1cells [59]. Since cytochalasin D did not inhibit the uptake of transferrin and ctx-b, strong evidence is provided that this inhibitor did not severely affect other endocytotic pathways as also observed by others [60]. A decreased uptake of 40 nm NPs and accumulation of NPs at the cell surface in the presence of cytochalasin D was observed. This could also be due to the formation of agglomerates on the cellular surface that would then show similar physio-chemical behavior as micron-size particles. Since accumulation at the cell border of 40 nm NPs was observed prior to actin-driven uptake, we argue that phagocytosis or macropinocytosis is involved in both the uptake of larger aggregates of 40 nm NPs and 1 µm particles. Our findings are in agreement with other studies that showed a reduced uptake of 40 nm carboxylated polystyrene particles in HeLa and 1321N1 cells in the presence of cytochalasin A [52] and by cytochalasin D in pulmonary macrophages [59]. In addition, the inhibition of 40 nm NP uptake also occurred in the presence of the clathrin inhibitor MDC in J774A.1 cells. This suggests that J774A.1 macrophages can employ multiple uptake mechanisms for the endocytosis of 40 nm PS NPs by both clathrin-mediated as well as macropinocytosis or phagocytosis. Caveolin-mediated uptake was not observed, since mβcd did not block the uptake of transferrin. Moreover, the related proteins such as caveolin-1 and flotillin-1 were not detected at the cell border. As already known, caveolin-mediated endocytosis is mainly observed in several cell types including capillary endothelium, type I alveolar epithelial cells, smooth muscle cells and fibroblasts [20]. Therefore, this result supports the cell type specific mechanism of this uptake.

The colocalization of clathrin heavy chain and the 40 nm PS NP fluorescence in the profile plots are also a strong hint for the involvement of a clathrin-mediated pathway in the uptake of 40 nm PS NPs by J774A.1. However, there were regions where 40 nm NPs did not colocalize with the clathrin heavy chain signal. The LSM data showed that J774A.1 cells express flotillin-1, and indeed profile plots of flotillin-1 have shown a colocalization with the 40 nm NPs [61]. It was shown that the phagosome proteome of J774A.1 cells contains high amounts of flotillin-1 [62]. However, all tested inhibitors for lipid raft-mediated uptake failed with this cell type. In addition, caveolin-1 was not detected. Therefore, the role of this uptake mechanism is still not clear in this cell and for this particle type. It also has been shown that caveolin-1 was only rarely present in human macrophages [62]. Consequently, one can conclude that NPs are taken up by a different mechanism in this cell type, which might also depend on the agglomeration behavior of particles on the cell surface, and thus the “secondary” size of the particles. Neither chlorpromazine nor mβcd (i.e., blocking the clathrin-mediated and the caveolin-mediated pathway) could fully inhibit the uptake of 40 nm NPs in A549 cells, since intracellular particle events could still be detected after treatment with those two inhibitors. Thus, A549 may also employ multiple endocytotic pathways, as it is known that different pathways can be used [52].

Finally, LSM was used (for exact spatial localization) to analyze the interaction of fluorescently labelled particles with single cells. This method is crucial since one can visually distinguish between extracellular and intracellular particle events. We also have attempted to quantify the uptake of the particles by fluorescence activated cell sorter (FACS). However, it was observed that the use of endocytotic pathway inhibitors resulted in particle agglomerates that were attached to the outer cell membrane which remained even after extensive washing. Therefore, FACS could not be used in combination with inhibitory conditions.

All particles internalized by endocytotic pathways are finally localized in intracellular vesicles. However, some other studies have shown that NPs of different materials were detected in the intracellular space and/or free in the cytoplasm. Since they were not membrane-bound, alternative uptake routes for cellular uptake might exist [22,46,59,63]. Possible uptake mechanisms such as receptor mediated diffusion via membrane pores as well as passive uptake by van der Waals or steric interactions (subsumed as adhesive interactions) [64–65] were proposed by the authors of these studies.

## Conclusion

It was shown that the particle size is critical in determining which endocytotic uptake route is deployed, and additionally, this process is cell type dependent. Not all inhibitors blocked the related pathway in the two different cell lines in the same way, which is also in agreement with the expected uptake mechanism per cell type. Therefore, each condition must be evaluated with the use of positive markers. A549 cells did not take up any 1 µm PS particles by phagocytosis/macropinocytosis over a period of 1 hour, whereas 40 nm NPs were ingested by clathrin- as well as caveolin-endocytotic pathways.

In J774A.1 cells, 1 µm particles were engulfed by phagocytosis/macropinocytosis and 40 nm PS NPs by both clathrin-mediated as well as macropinocytosis or phagocytosis. Therefore, it seems that both cell types take up NPs by different mechanisms, although the mechanisms are not similar for the different cell types.

## Experimental

### Cell cultures

Human alveolar epithelial type II cells (A549 cell line) and mouse macrophage cells (J774A.1 cell line), both from American tissue Type Culture Collection, were cultured in RPMI 1640 with HEPES (Gibco, Luzern, Switzerland) completed with 10% foetal bovine serum (heat inactivated, PAA Laboratories, Austria), 1% L Glutamine (Gibco, Luzern, Switzerland) and 1% penicillin/streptomycin (Gibco, Luzern, Switzerland) and kept at 37 °C and 5% CO_2._ The A549 epithelial cells were split twice per week using trypsin (0.05% trypsin-EDTA, GIBCO, Switzerland). J774A.1 cells were sub-cultured using the scraping method, resuspended in the medium and finally centrifuged (2 min, 2000 rpm and 21 °C). A549 cells were seeded at a density of 35 × 10^3^ cells/mL and J774A.1 cells at a density of 25 × 10^4^ cells/mL in BD FalconTM 4 chamber polystyrene vessels with tissue culture treated glass slide with a growth area of 1.7 cm^2^ (Milian, Geneva, Switzerland). A549 cells were grown to confluence for 7 days prior to exposure experiments and J774A.1 cells for 1 day and allowed to adhere prior to use.

### Particle characterization and exposure

The commercially available carboxylate Fluoresbrite™ plain yellow green (cataloged as 1 µm hydrodynamic diameter) and red (cataloged as 40 nm hydrodynamic diameter) polystyrene (PS) particles (Molecular Probes, Luzern, Switzerland) were used for the study. The particles were characterized in terms of size by dynamic light scattering (DLS) and transmission electron microscopy (TEM). The hydrodynamic radius was acquired by DLS (3D LS Spectrometer, LS Instruments AG, Fribourg, Switzerland) based on a viscosity of 0.89 mPas and a refractive index of 1.33. The correlation function was measured at a scattering angle of 90° at *T* = 25 °C. The data was analyzed with a fitting routine applying a single exponential model and accounting for polydispersity assuming a Schulz–Zimm distribution. Both particles were measured at a concentration of 20 µg/mL in either unsupplemented RPMI medium or dH_2_O over a time window of 1 hour.

The particles were observed with a Hitachi transmission electron microscope (H-7100, Tokyo, Japan) operating at an acceleration voltage of 75 kV and equipped with a Morada CCD digital camera (Olympus, Tokyo, Japan). Size distributions of both PS particle types were obtained by image analysis using Fiji software. Thereby, images were converted to binary images by automated thresholding (default threshold) and analyzed applying an ellipse fit. A total of 982 counts were performed for the NPs and 102 counts were collected for the micron-sized particles. The electrophoretic mobility of the particles was determined by electrophoresis (Brookhaven 90 Plus Instruments Corp., Holtsville, USA). The measured particle mobility in the electric field was transformed to the zeta potential by applying the Smoluchowski model. The small particles were measured at a concentration of 60 µg/mL and the larger particles at 20 µg/mL.

Prior to the cell exposure, all particle suspensions were sonicated for 2 minutes in order to avoid aggregation. Polystyrene particles were suspended in RPMI 1640 medium and adjusted to a concentration of 20 µg/mL. A 1 mL particle suspension was applied on a growth area of 1.7 cm^2^ and the cells were exposed for 1 hour in the well.

### Endocytotic uptake proteins

Antibodies against clathrin heavy chain, flotillin-1 and caveolin-1 (all fluorescently labelled with Alexa Fluor 488, antibodies-online GmbH, Aachen, Germany) were used at a final dilution of 1:20 in 1× PBS on fixed cells. After 1 hour of staining (in a dark room at room temperature) and three washing cycles with 1× PBS, the cells were mounted using medium Glycergel mounting medium (C0563, Dako, Baar, Switzerland). The intensity profiles were performed using Fiji software. The pearson coefficient (r_p_) reavealed the colocalization/signal overlap of endocytotic uptake proteins and NPs for all analyzed regions.

### Inhibition of endocytosis

Different endocytotic inhibitors were tested for their optimal concentration, exposure time and cell impairment (Table 1, Figure 3). Inhibition of clathrin-mediated endocytosis in both cell types was tested with chlorpromazine hydrochloride (C8138, Sigma-Aldrich, Switzerland) and monodansylcadaverine (Dansylcadaverine, D4008, Sigma-Aldrich). Inhibition of caveolin-mediated endocytosis was performed in both cell lines using 10 mM methyl-β-cyclodextrin (mβcd) (C4555, Sigma-Aldrich). A 4 µM cytochalasin D (C8273, Sigma-Aldrich) solution was used to inhibit phagocytosis and macropinocytosis in both cell lines.

### Cell treatment

Both cell types were preincubated for 30 minutes and subsequently exposed to either the control substances (i.e., transferrin or ctx-b) or the PS particles, or in combination with the inhibitor (for continuous inhibition). Exposures were carried out for 1 hour.

### Control experiments

Alexa fluor 488 coated transferrin (T13342, Invitrogen), dissolved in dH_2_O containing 1% NaN_3_ and diluted in RPMI to a final concentration of 120 μg/mL, was used to test the inhibition of clathrin-mediated endocytosis. Cholera toxin subunit-b (ctx-b) labelled with Alexa Fluor 488 conjugate (C34775, Molecular Probes) was used to test inhibition of caveolin-mediated endocytosis. Ctx-b was dissolved in 10 mM phosphate-buffered saline (PBS, pH 7.4) containing 1% NaN_3_ and diluted to a final concentration of 0.6 μg/mL 1× PBS (pH 7.4) in RPMI prior to use. To test macropinocytosis and phagocytosis, 1 µm carboxylate modified fluospheres (molecular probes) were used at a concentration of 20 µg/mL in RPMI.

### Laser scanning microscopy of fixed and living cells

For LSM imaging, the cells were fixed with 3% paraformaldehyde (PFA, Sigma-Aldrich, Switzerland) in PBS for 15 minutes at room temperature. The cells were then washed with 1× PBS, then permeabilized for 15 minutes with 0.2% Triton X and then washed again with 1× PBS. The F-actin cytoskeleton was stained with rhodamine phalloidin (Invitrogen, Luzern, Switzerland) at a dilution of 1:50 in 1× PBS. Following this step, the cells were then washed and mounted in glycergel mounting medium (C0563, Dako, Baar, Switzerland). Image acquisition was performed with an inverted Zeiss LSM 710 Meta (Axio Observer.Z1, Zeiss, Switzerland) microscope equipped with 405, 488 and 561 nm laser excitation sources. Fields of interests were acquired at 5 different randomly selected areas per glass slide. The experiment was performed using a 63x/N.A 1.4 immersion oil lens. Cellular and morphological information was retrieved using Imaris software (Bitplane 7.4, Zürich, Switzerland).

For live cell imaging, the cells were seeded in a Lab-Tek^TM^ II chambered coverglass 4 chamber well (1.5 german coverglass system, NC-155382, Nunc, Milian, Geneva, Switzerland), stained with Cell tracker^TM^ violet BMQC dye and incubated for 1 hour at 37 °C and 5% CO_2_ followed by three washing steps with 1× PBS. Finally, transparent RPMI 1640 medium (no 1% L-glutamine, no antibiotics, no fetal calf serum and without phenol red pH indicator) was added with either 40 nm or 1 µm polystyrene particles alone or in combination with the inhibitor, and time lapse imaging was started. The live cell imaging ran over a time period of 60 minutes during which the cells were kept in a constant environmental at 37 °C and 5% CO_2_. Image acquisition was performed as described above.

### Lactate dehydrogenase (LDH) assay

A 1 mL sample of the supernatant of each experiment was collected and stored at 4 °C to determine cytotoxicity. Triton X (0.2% in unsupplemented RPMI) was used for cell lysis as a positive control. The supernatant of untreated cells was used as negative control. The LDH assay was performed with the Cytotoxicity Detection Kit (Roche Applied Science, Mannheim, Germany) according to the supplier's manual. Each supernatant was measured in triplicate. All measurements were analyzed as *n* = 3 experiments.

### Trypan blue exclusion assay

The assay was carried out according to the manufacturer’s manual (Sigma-Aldrich, Steinheim, Germany). Trypan was added to untreated cells and to cells which showed impairment by the inhibitors (Table 1). At a dilution of 1:2 in trypan blue, the cells were stained and counted in a Neubauer chamber (Blau Brand, Ref. 717805, Wertheim, Germany). The positive control was performed by adding 0.2% Triton X to the cells for 5 minutes, prior adding trypan blue. All measurements were analyzed as *n* = 3 experiments.

## Supporting Information

File 1 Lactate dehydrogenase (LDH) assay and live laser scanning microscopy measurements which reveal particle uptake in J774A.1 and A549 cells.
