# Supplementary material for: Different endocytotic uptake mechanisms for nanoparticles in epithelial cells and macrophages
Source: Beilstein J Nanotechnol. 2014 Sep 24;5:1625–36. doi: 10.3762/bjnano.5.174 (PMC4222452; doi:10.3762/bjnano.5.174)
Supplement: File 1 — Lactate dehydrogenase (LDH) assay and live laser scanning microscopy measurements which reveal particle uptake in J774A.1 and A549 cells. [file Beilstein_J_Nanotechnol-05-1625-s001.pdf]

Supporting Information

for

Different endocytotic uptake mechanisms for nanoparticles in epithelial cells and macrophages

Dagmar A Kuhn[1], Dimitri Vanhecke[1], Benjamin Michen[1], Fabian Blank[2], Peter Gehr[3], Alke Petri-Fink[1], Barbara Rothen-Rutishauser*[1,2]

Address: [1]Adolphe Merkle Institute, University of Fribourg, Chemin des Verdiers 4, 1700 Fribourg, Switzerland, Phone +41 26 300 95 02, [2]Respiratory Medicine, University Hospital of Bern, Murtenstrasse 50, 3008 Bern, Switzerland and [3]Institute of Anatomy, University of Bern, Baltzerstrasse 2, 3000 Bern 9, Switzerland

Email: Barbara Rothen-Rutishauser* - barbara.rothen@unifr.ch

*Corresponding Author

Lactate dehydrogenase (LDH) assay and live laser scanning microscopy measurements to reveal particle uptake in J774A.1 and A549 cells

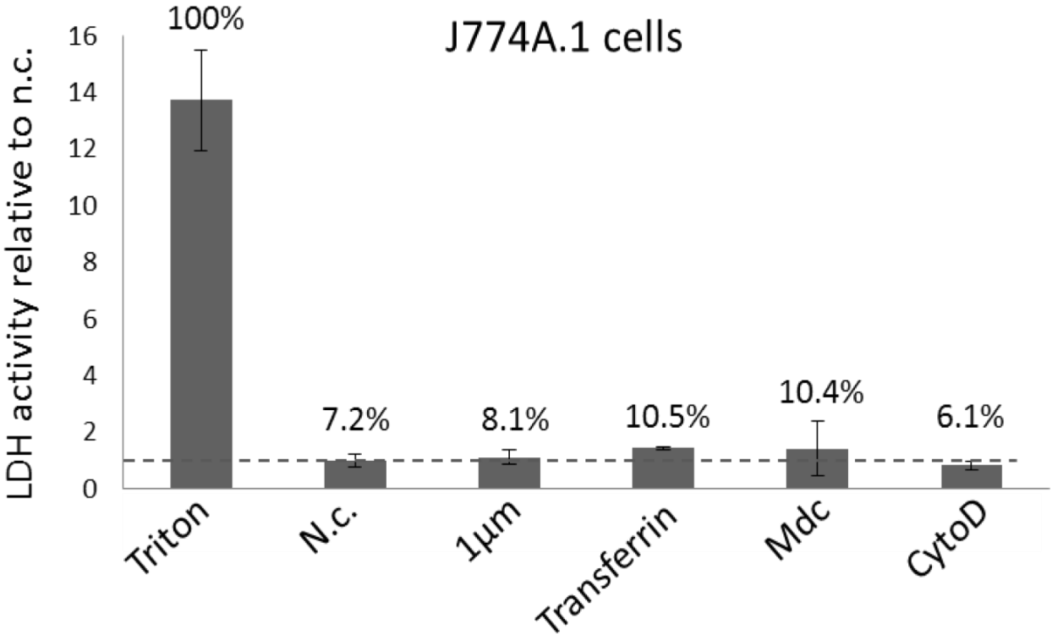

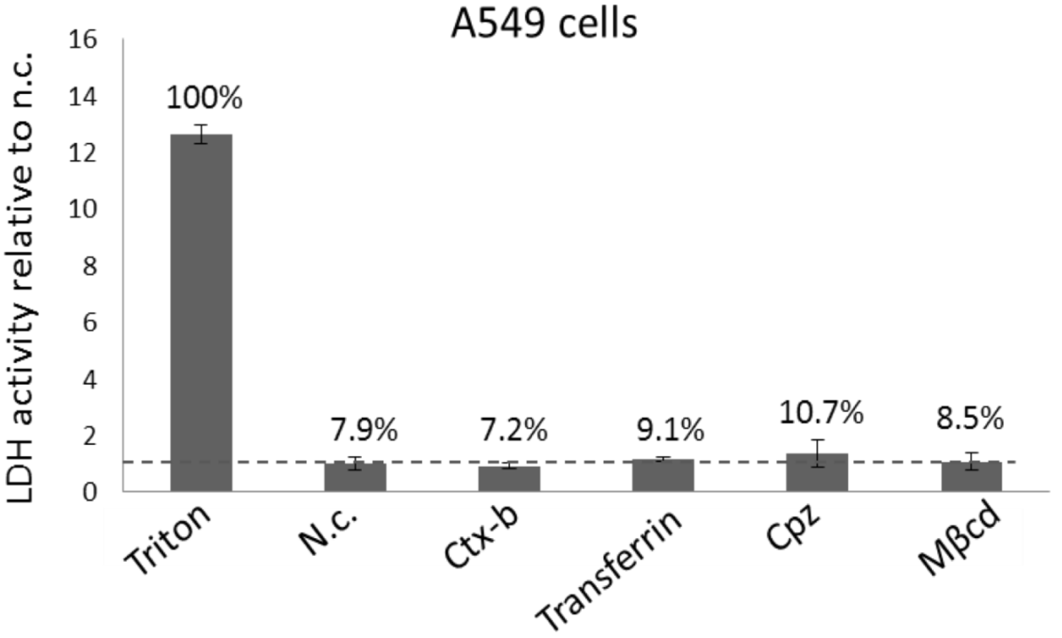

Figure S1: Lactate dehydrogenase (LDH) assay. Description: LDH activity was measured with respect to the negative control for both cell types and percentages show the measured samples compared to the positive control (triton). Neither the A549 cells, nor the J774A.1 cells showed an increased value for concentrations tested. These concentrations were used for both the inhibition and uptake studies for fixed and living cells. All measurements were performed in triplicate for all samples.

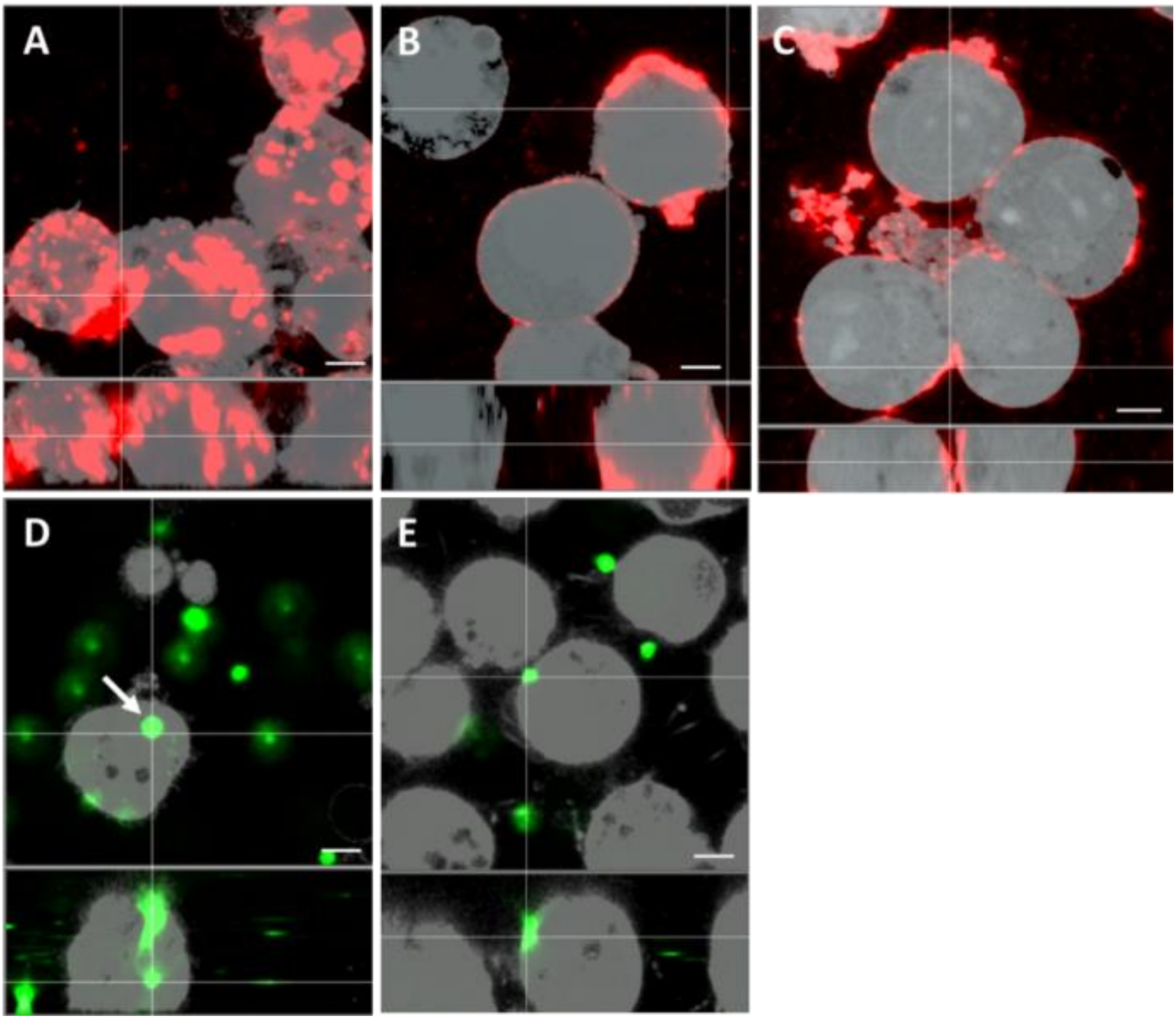

Figure S2: Live laser scanning microscopy measurements to reveal particle uptake in J774A.1 cells. Description: (A) Untreated cells with 40 nm NPs. (B) 40 nm NPs and monodansylcadaverine. (C) 40 nm NPs and cytochalasin D. (D) Untreated cells with 1 µm particles. (E) 1 µm particles and cytochalasin D. NP: Nanoparticle, scale bar: 5 µm. 40 nm NPs are shown in red and 1 µm particles are shown in green. Cytosol: grey

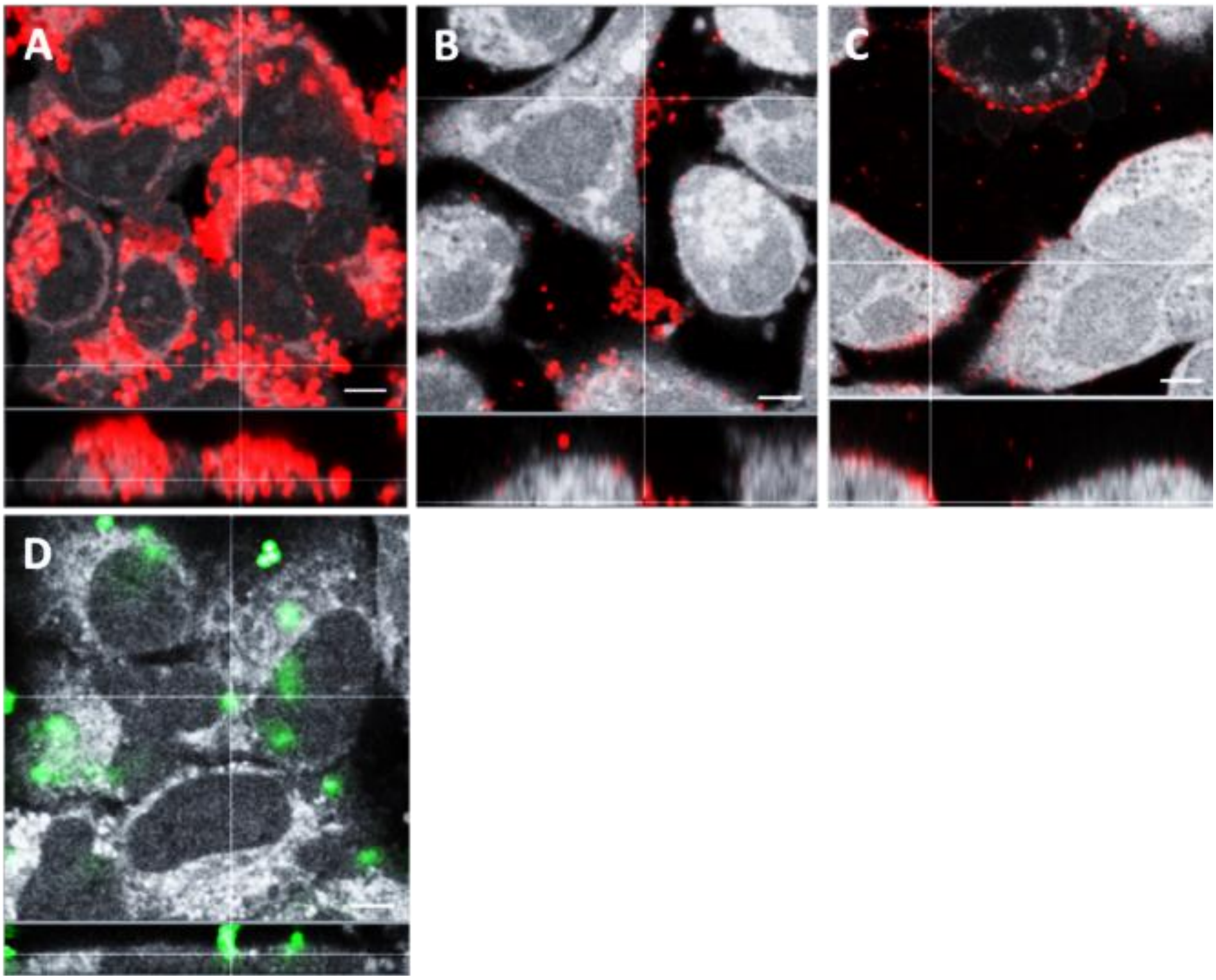

Figure S3: Live laser scanning microscopy measurements to reveal particle uptake in A549 cells. Description: (A) Untreated cells with 40 nm NPs. (B) 40 nm NPs and chlorpromazine. (C) 40 nm NPs and mβcd. (D) Untreated cells with 1 µm particles. mβcd: methyl-β-cyclodextrin; NP: Nanoparticle, scale bar: 5 µm. 40 nm NPs are shown in red and 1 µm particles are shown in green. Cytosol: grey
